# Who’s in and Who’s Out? Selection Bias in Aging Research

**DOI:** 10.1093/geroni/igaa057.2998

**Published:** 2020-12-16

**Authors:** Elizabeth Rose Mayeda, Eleanor Hayes-Larson, Hailey Banack

**Affiliations:** 1 University of California Los Angeles, Los Angeles, California, United States; 2 University of Buffalo, Buffalo, New York, United States

## Abstract

Selection bias presents a major threat to both internal and external validity in aging research. “Selection bias” refers to sample selection processes that lead to statistical associations in the study sample that are biased estimates of causal effects in the population of interest. These processes can lead to: (1) results that do not generalize to the population of interest (threat to external validity) or (2) biased effect estimates (associations that do not represent causal effects for any population, including the people in the sample; a threat to internal validity). In this presentation, we give an overview of selection bias in aging research. We will describe processes that can give rise to selection bias, highlight why they are particularly pervasive in this field, and present several examples of selection bias in aging research. We end with a brief summary of strategies to prevent and correct for selection bias in aging research.

